# Customizing droplet contents and dynamic ranges via integrated programmable picodroplet assembler

**DOI:** 10.1038/s41378-019-0062-5

**Published:** 2019-07-01

**Authors:** Pengfei Zhang, Aniruddha Kaushik, Kuangwen Hsieh, Tza-Huei Wang

**Affiliations:** 1 Department of Biomedical Engineering, Johns Hopkins University, 3400 N. Charles Street, Baltimore, MD 21218 USA; 2 Department of Mechanical Engineering, Johns Hopkins University, 3400 N. Charles Street, Baltimore, MD 21218 USA

**Keywords:** Engineering, Nanoscale devices

## Abstract

Droplet microfluidic technology is becoming increasingly useful for high-throughput and high-sensitivity detection of biological and biochemical reactions. Most current droplet devices function by passively discretizing a single sample subject to a homogeneous or random reagent/reaction condition into tens of thousands of picoliter-volume droplets for analysis. Despite their apparent advantages in speed and throughput, these droplet devices inherently lack the capability to customize the contents of droplets in order to test a single sample against multiple reagent conditions or multiple samples against multiple reagents. In order to incorporate such combinatorial capability into droplet platforms, we have developed the fully Integrated Programmable Picodroplet Assembler. Our platform is capable of generating customized picoliter-volume droplet groups from nanoliter-volume plugs which are assembled in situ on demand. By employing a combination of microvalves and flow-focusing-based discretization, our platform can be used to precisely control the content and volume of generated nanoliter-volume plugs, and thereafter the content and the effective dynamic range of picoliter-volume droplets. Furthermore, we can use a single integrated device for continuously generating, incubating, and detecting multiple distinct droplet groups. The device successfully marries the precise control and on-demand capability of microvalve-based platforms with the sensitivity and throughput of picoliter droplet platforms in a fully automated monolithic device. The device ultimately will find important applications in single-cell and single-molecule analyses.

## Microfabrication: new platform to produce customized picodroplets

Researchers in the United States have developed a system to produce tunable groups of picoliter-scale droplets (picodroplets) with customized contents. A team led by Tza-Huei Wang of Johns Hopkins University designed and tested the Integrated Programmable Picodroplet Assembler, which combines microvalves and flow focusing to enable the dynamic tuning of picodroplet number and content. Programmable microvalves are used to combine samples, reagents, and buffers into nanodrops. These nanodrops are then propelled through a flow-focusing junction which divides them into a specific number of picodroplets. Picodroplet content and picodroplet number can be dynamically tuned by adjusting the microvalves. This new approach combines the precision and on-demand control of microvalves with high throughput, offering researchers a tunable system to assemble multiple samples or reagents for clinical or industrial applications.

## Introduction

Droplet microfluidics has recently emerged as an attractive technological platform for high-throughput and high-sensitivity biochemical analysis^1–3^ with applications ranging from drug screening^4,5^, directed evolution^6^, antibody screening^7^, and protein crystallization^8^, to disease genotyping^9,10^ and antibiotic susceptibility testing (AST)^11–13^. This burgeoning interest in droplet microfluidics can be attributed to its many advantages. First of all, by confining assay reactants into discrete femtoliter to picoliter reaction volumes, droplets facilitate a significant reduction in the assay background and therefore help in drastically improving the signal-to-background ratio and the overall detection sensitivity in bioassays^10,14–16^. Due to the decreased volume of each reaction, droplets can significantly reduce the amounts of reagents consumed per reaction, resulting in significant cost savings compared with bulk reactions. Furthermore, most droplet platforms can be operated passively and generate droplets at kilohertz frequencies or above, offering an easier and faster way to perform massively parallel reactions compared with conventional microtiter plates.

Despite their many advantages, current droplet technologies are limited to generating droplets consisting of a single sample subject to a homogeneous or random reagent condition. Many applications, however, require the ability to analyze a set of samples against a panel of reagents (often at a series of dilutions) in a “combinatorial” manner. For example, although droplet digital PCR platforms have become a turnkey tool for quantitative detection of rare biomarkers such as genetic mutations^9^, detection of each additional target via droplet digital PCR would require an additional device or complex optical indexing schemes^9,17–20^. Methods such as picoinjection^21–23^ and droplet merging^24,25^ represent early attempts toward achieving combinatorial analysis within a single droplet device by adding a single reagent at a single concentration into droplets. To achieve more controlled customization of droplet content, Kaminski et al. developed a platform that coupled capillary delivery of plug sequences with droplet generation; however, this platform generated relatively large droplet volumes (~nL) and still required manual assembly off-chip or external valves to tune the droplet content^26^. In a more recent demonstration, Chang et al. developed a flow-focusing-based droplet generation device with programmable pneumatic valves that produced droplets with custom contents^27^. Unfortunately, as this device functioned merely as a droplet generator, this platform necessitated user intervention to transport each droplet group off-chip for incubation and detection in a “fragmented” workflow. Such a fragmented workflow offers flexibility for modifying assay conditions, but the manual collection and transfer steps can cause unwanted droplet merging and loss. Hence, a fully integrated droplet platform capable of achieving combinatorial analysis remains in high demand.

In response, we have developed the Integrated Programmable Picodroplet Assembler (iPPA), a fully integrated device for achieving on-demand production, incubation, and detection of multiple groups of picodroplets with a tunable dynamic range (i.e., picodroplet number) and programmable control of content (i.e., reaction condition). We accomplish this by first coupling the on-demand controllability of pneumatic microvalve-based droplet platforms^28–30^ with the high sensitivity and high throughput of picodroplet platforms. We then incorporate on-chip incubation and fluorescence detection modules, enabling the iPPA device to achieve (1) in situ assembly of samples, reagents, and buffers into nanoliter plugs (i.e., nanoplugs) with distinct reaction volumes, combinations, and dilutions, (2) mixing of nanoplugs, (3) discretization of the assembled nanoplugs into picodroplets, (4) picodroplet incubation, and (5) in-line fluorescence detection. To demonstrate the convenience and utility of our iPPA platform, we generate multiple groups of monodisperse picodroplets with customized dynamic ranges and uniform contents—all in automation without manual intervention.

## Results

### Overview and operation of iPPA

**Fig. 1:**
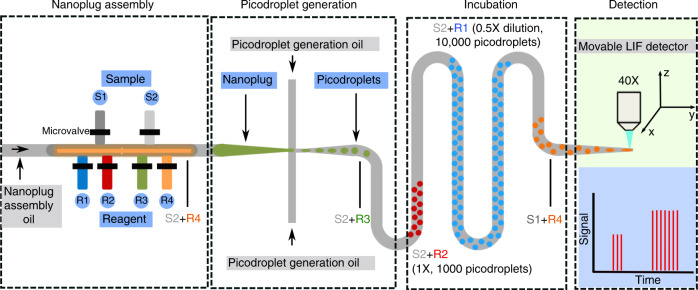
Schematic of the iPPA platform. On-demand generation of picodroplets with tunable dynamic ranges and customized contents is achieved by assembling nanoplugs with programmable size and customized mixtures of samples and reagents. Nanoplug assembly via microvalves, picodroplet generation via flow focusing, incubation, and detection via laser-induced fluorescence (LIF) are all integrated in a single iPPA device

On-demand generation of picodroplets with a tunable dynamic range and precise control of picodroplet content in our iPPA device is achieved by assembling nanoplugs with programmable size and content and subsequently discretizing each nanoplug into monodisperse picodroplets (Fig. 1). In the nanoplug assembly module of our device, we use programmable microvalves to combine samples, reagents, and buffers into nanoplugs at various total volumes (i.e., sizes) and volume ratios (i.e., contents). The assembled nanoplugs are propelled by pressurized nanoplug assembly oil into a flow-focusing junction, where the picodroplet generation oil continuously discretizes nanoplugs into monodisperse picodroplets. Consequently, each nanoplug of a preprogrammed size and content produces a fixed number of picodroplets with the same content. Once produced, each group of picodroplets is propelled by the picodroplet generation oil to travel through the incubation channel and finally a detection constriction, where each picodroplet is sequentially detected using laser-induced fluorescence (LIF). Fluorescence detection of individual picodroplets in sequence yields a time trace of fluorescence intensities, thus allowing the characterization of the number of picodroplets in each group, as well as their sizes and contents.

**Fig. 2:**
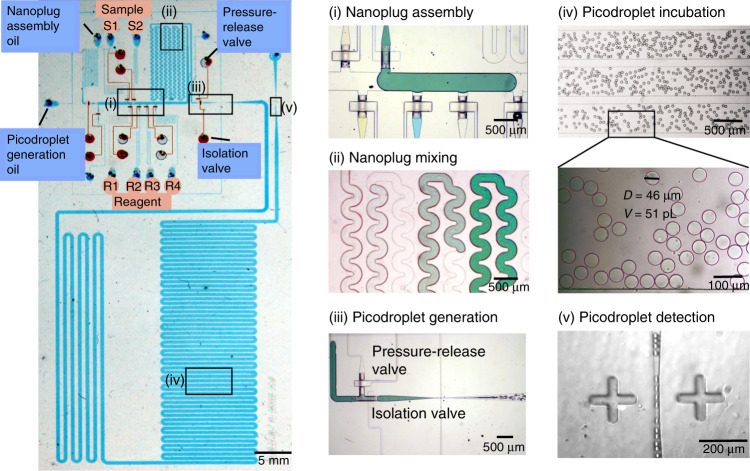
Integration of multiple operations in the iPPA device. The integrated iPPA PDMS device, which consists of a valve layer (red) that controls flow in a fluidic layer (blue), integrates several important functions vital to a programmable picodroplet assembly. In the device, (i) reaction solutions are assembled into nanoplugs via PDMS microvalves; (ii) nanoplugs are mixed in a serpentine channel; (iii) picodroplets are generated through a flow-focusing junction; (iv) picodroplets are incubated in an incubation channel where they remain intact and monodisperse (average diameter: 46 µm and average volume: 51 pL (inset)); and finally, (v) picodroplets funnel through the detection site where the fluorescence of each picodroplet can be measured by a LIF detector

We designed several features in our iPPA device to achieve a programmable and highly robust picodroplet assembly (Fig. 2). iPPA uses a two-layer architecture^31,32^ where microvalves in the valve control layer (Fig. 2, red) regulate the fluid flow and hence the operation of five modules in the fluidic layer (Fig. 2, blue): nanoplug assembly, nanoplug mixing, picodroplet generation, picodroplet incubation, and picodroplet detection (Fig. 2, insets). In the nanoplug assembly module, a nanoplug assembly oil inlet, two sample inlets, and four reagent inlets feed into the central channel and facilitate the assembly of nanoplugs in this channel. The nanoplug assembly oil, samples, and reagents are pre-loaded into their respective inlets and pressurized so that they can be injected into the central channel and assembled into nanoplugs when their respective microvalves are opened (Fig. 2(i)). Once assembled, the nanoplugs are propelled by the nanoplug assembly oil into the serpentine mixing channel to enhance chaotic mixing^33^ within the nanoplugs (Fig. 2(ii)). Between the mixing channel and the flow-focusing junction, we have added an “isolation” valve and a pressure-release valve to help maintain robust assembly of nanoplugs and discretization of nanoplugs through the 10-µm-wide flow-focusing junction into ~50-pL picodroplets (Fig. 2(iii)). We space the generation of adjacent groups of picodroplets such that they remain spatially separated from each other while flowing through the incubation channel (Fig. 2(iv)). The picodroplets finally funnel through the 10-µm-wide detection constriction (Fig. 2(v)) to facilitate fluorescence detection of individual picodroplets. While in operation, our entire iPPA device is interfaced with a supporting thermo-optical instrument for incubation temperature control and on-chip fluorescence measurements (Fig. S1). Specifically, a programmable thermoelectric heater posits underneath the incubation region of the iPPA device. Fluorescence detection is achieved with a custom LIF detector, which uses a 552-nm laser for excitation and a silicon avalanche photodiode (APD) for detection. Notably, our LIF detector is equipped with a three-axis movable stage which allows us to easily measure droplet fluorescence at any location on our iPPA device.

### Flexible nanoplug assembly with precise control of size and content

**Fig. 3:**
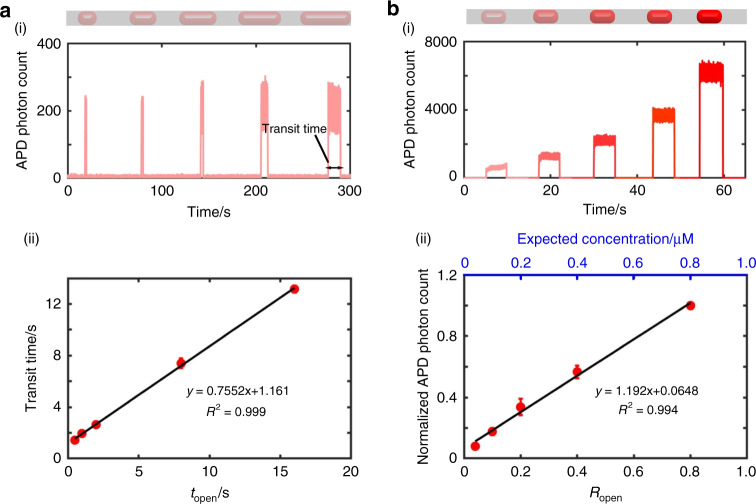
Programmable nanoplug assembly in the iPPA device. We programmatically control (**a**) the size and (**b**) the content of nanoplugs by modulating the microvalve opening time (*t*_open_) and ratio of microvalve opening time for fluorescent DNA (*R*_open_). **a** (i) The fluorescence trace of the five nanoplugs assembled with increasing *t*_open_ shows increasing transit times, which are used to characterize the nanoplug size. APD photon count indicates the number of incident photons detected per 0.1 ms by a silicon avalanche photodiode detector. **a** (ii) The nanoplug size, denoted by its transit time, increases linearly with *t*_open_ (*R*^2^ = 0.999). **b** (i) The fluorescence trace of the five nanoplugs assembled with increasing (*R*_open_) shows increasing fluorescence intensities. **b** (ii) The average normalized nanoplug fluorescence intensities increase linearly with *R*_open_ (*R*^2^ = 0.994). All results were measured from three different experiments. The error bars in **a** (ii) and **b** (ii) depict ± 1 standard deviation

We are able to precisely control the volume of assembled nanoplugs in our device by modulating the opening times of the microvalves controlling the sample and reagent inlets. To demonstrate this, we assembled five nanoplugs of increasing volumes by programming increasing valve opening times for sample (1 µM solution of a fluorescently labeled single-stranded DNA probe) and reagent (phosphate-buffered saline (PBS)) inputs. During nanoplug assembly, we increased the combined opening times of the two microvalves (denoted hereafter as *t*_open_) at 0.5, 1, 2, 8, and 16 s but kept the ratio of the opening times between the two microvalves (denoted hereafter as *R*_open_) constant to ensure equal DNA concentrations across all nanoplugs. In order to characterize the content and volumes of our generated nanoplugs, we measured the fluorescence signals emitted by each nanoplug, as they sequentially passed by our LIF detector, which we positioned immediately after the mixing region. From the resulting time trace of fluorescence signals, we observe five peaks with increasing peak widths, which is indicative of five nanoplugs of increasing volumes (Fig. 3a(i)). Notably, the heights of all five peaks are approximately equal, indicating nearly equal concentrations of fluorescent DNA within the nanoplugs. We measured the width of each fluorescent peak to determine the transit time of each nanoplug. The transit time is proportional to the volume of each nanoplug and provides an effective surrogate for measuring nanoplug volumes. The average transit time of nanoplugs increases linearly with the associated *t*_open_ in three distinct devices (Fig. 3a(ii)). The strong linearity (*R*^2^ = 0.999) and small error bars in our plot demonstrate the reliability of our microvalves in precisely controlling the volume of injected solutions on demand. These results are consistent with previous observations from similar microvalve-based devices^30^.

We are also able to achieve precise control of nanoplug content within our iPPA device. To demonstrate this, we assembled five nanoplugs with identical volume (by fixing *t*_open_ = 2.5 s) but with increasing concentrations of fluorescently labeled DNA in PBS by increasing *R*_open_ at 0.04, 0.1, 0.2, 0.4, and 0.8. Following nanoplug assembly and mixing, the nanoplugs were again sequentially detected using our LIF detector at the end of the mixing channel. In the resulting time trace, these nanoplugs produced fluorescent peaks with similar widths but increasing heights, indicating that these five nanoplugs indeed had similar sizes but increasing concentrations of fluorescent DNA (Fig. 3b(i)). As expected, the average fluorescence intensity of nanoplugs increases linearly with *R*_open_ in three distinct devices (Fig. 3b(ii)). The strong linearity (*R*^2^ = 0.994) and the small error bars in our plot demonstrate the capability and reliability of our valve actuation to program the nanoplug content on demand. These results are also consistent with previous observations in similar nanoplug assembly devices^30^. While we demonstrate herein up to 25-fold dilution of our sample, we note that larger dilution factors are achievable by simply increasing the size of the assembled nanoplug or by serial dilution using all five inlets of our device.

### Stable assembly of nanoplugs and monodisperse generation of picodroplets

A key feature of our iPPA device is the isolation valve (Fig. 2(iii)), which ensures stable and reproducible nanoplug assembly. In our device, we continuously inject the picodroplet generation oil through the flow-focusing junction into the incubation region. This continuous stream of oil not only creates a constant backpressure during nanoplug assembly, but the oil also has a different surfactant composition from the nanoplug assembly oil and can destabilize nanoplugs upon direct contact. We therefore add the isolation valve between the nanoplug mixing region and the flow-focusing junction and close it during nanoplug assembly to prevent the picodroplet generation oil from back-flowing into the nanoplug assembly and mixing regions and disrupting nanoplugs. The isolation valve can also shield picodroplets in the incubation region from potential pressure perturbations during nanoplug assembly. The isolation valve works in tandem with a pressure-release valve. When the isolation valve is closed, the pressure-release valve is opened to atmosphere to allow nanoplugs to flow through the mixing region. As nanoplugs approach the flow-focusing junction, the pressure-release valve is closed and the isolation valve is opened, thus allowing the nanoplugs to be discretized into picodroplets.

**Fig. 4:**
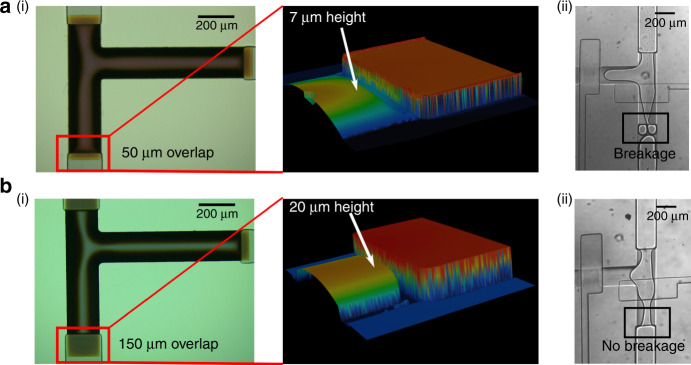
Optimizing channel design for smooth transition from nanoplugs to picodroplets. **a** (i) When the transition region between the mixing channel and isolation channel is too shallow (~7 µm), (ii) nanoplugs undergo severe breakage during flowing through which it results in droplet size variation; **b** after the height of the transition region was increased to 20 µm by increasing SPR220 channel height and after enlarging overlap between SPR220 channel and SU-8 channel from 50 to 150 µm (i), nanoplug breakage was greatly reduced and (ii) smooth transition from nanoplugs to picodroplets was guaranteed

We optimized the design of the channel that resides underneath the isolation valve (isolation channel) to facilitate smooth transition of nanoplugs into picodroplets. We found that it is essential to create a smooth transition region between the mixing channel and the isolation channel for smooth and uninterrupted flow of nanoplugs, which is necessary to guarantee stable picodroplet generation. Indeed, we observed that if the transition region between the mixing channel and the isolation channel is too shallow (~7 µm) (Fig. 4a(i)), the abrupt constriction can cause nanoplugs to break (Fig. 4a(ii)), inducing fluctuations in picodroplet size. In contrast, if the height of the transition region height is increased from 7 to 20 µm (Fig. 4b(i)), nanoplug breakage can be obviated, and we can obtain smooth flow in transitioning from nanoplugs to picodroplets (Fig. 4b(ii)).

We also determined the pressure for driving nanoplugs through the flow-focusing junction to achieve optimal picodroplet monodispersity. We first generated a nanoplug containing a fixed concentration of fluorescent DNA in the assembly region of our device, and then pushed it past the isolation valve into the flow-focusing junction at 4.7 psi. The generated picodroplets flowed through the droplet incubation region and then the detection constriction, where picodroplets were individually measured for fluorescence by our LIF detector. This resulted in a fluorescence time trace in which each peak represents a traversing picodroplet (Fig. S2A). As a surrogate for droplet volume, we measured the width of the droplet peak, or droplet transit time (Fig. S2B), for each picodroplet. We measured transit times of picodroplets from four more droplet groups by repeating this experiment four more times, increasing the nanoplug driving pressure by 0.5 psi each time. In order to characterize the monodispersity of each picodroplet group, we calculated the coefficient of variation (CV) of droplet transit times in each picodroplet group and plotted it against the driving pressure used to generate droplets in that group (Fig. S3). We performed this experiment and subsequent analysis in triplicate using three distinct devices. At 5.2 psi, we generate the most monodisperse picodroplet group with the least chip-to-chip variation.

### Customized dynamic ranges of picodroplet groups

**Fig. 5:**
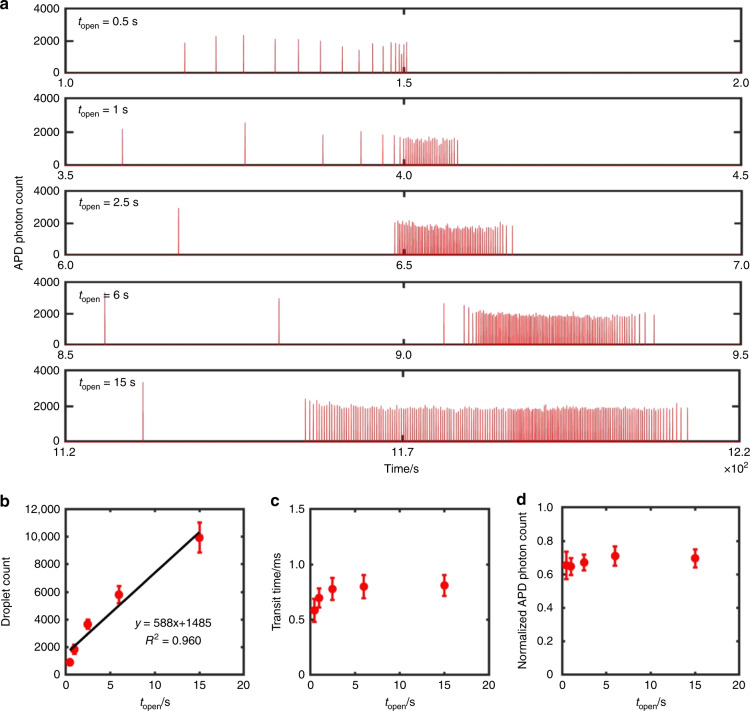
Customized dynamic ranges of picodroplet groups in iPPA. **a** The fluorescence time trace of the five picodroplet groups with increasing *t*_open_ shows increasing droplet counts. APD photon count indicates the number of incident photons detected per 0.1 ms by a silicon avalanche photodiode detector. **b** The number of droplets in each group increases linearly with *t*_open_ (*R*^2^ = 0.960) and serves as a standard curve to assist users with selection of an appropriate dynamic range. **c** The droplet volume, characterized by the droplet transit time, remains approximately equal with increasing *t*_open_. **d** All results were measured from three different experiments. The error bars in **b**–**d** depict ± 1 standard deviation

The ability to control the volume of nanoplugs in our device allows us to customize the number of picodroplets per nanoplug. This capability is useful for customizing the experimental dynamic range for each nanoplug and its unique testing condition; that is, iPPA offers the flexibility to analyze fewer picodroplets for reducing assay turnaround time and conserving reagents or to analyze more picodroplets for detecting rare targets and performing replicates. We demonstrate this capability by assembling five nanoplugs containing the same concentration of fluorescent DNA but with increasing volumes by increasing *t*_open_ (0.5, 1, 2.5, 6, and 15 s), and detecting each resulting group of picodroplets at the end of the incubation channel. To isolate groups of picodroplets, we waited for each nanoplug to completely discretize into picodroplets before we assembled the next nanoplug. The resulting time trace clearly shows five separate groups of picodroplets with an increasing number of picodroplets in each group (Fig. 5a; displaying 1 in every 50 picodroplets from each droplet group for ease of viewing). Clean separation between adjacent groups of picodroplets offers a means to identify each group even when picodroplets have similar fluorescence intensities. The iPPA device ensures clear separation between adjacent droplet groups with as little as 5 min of spacing between the assembly and processing of each group. When operated continuously, the iPPA device can readily process 12 unique droplet groups per hour, which is a vast improvement over the capabilities of conventional platforms. Finally, we point out that, besides pre-adjusting the driving pressures for the device, the process of generating nanoplugs and subsequent picodroplets was completely automated by our MATLAB program without any operator interference.

Upon inspection of the entire droplet fluorescence trace, we note that the majority of droplets in each droplet group have similar intensities, though a small fraction of droplets in the beginning of each group have higher intensities (Fig. S4, left). These early, loosely packed picodroplets in each group were likely generated before picodroplet discretization from each nanoplug reached steady-state equilibrium and were more subjected to Taylor dispersion^34^. By contrast, the trailing droplets of each group were likely generated at a steady state and were more tightly packed, resulting in minimal dispersion. By superimposing a histogram of droplet frequency (1-s bins) over the droplet fluorescence trace, we clearly demonstrate that these sparse droplets with higher intensities indeed account for only a small fraction of each droplet group (Fig. S4, right) and therefore pose a negligible impact on the performance of our platform.

To evaluate the capabilities of our iPPA device, we characterize the effect of the dynamic range on volume monodispersity and uniformity of picodroplet content. We first generated a standard curve to assist users with selection of an appropriate dynamic range or droplet number per group. Specifically, from triplicate experiments, we plotted the numbers of picodroplets generated against *t*_open_ used to assemble their respective nanoplug (Fig. 5b). Here, we can clearly see that the number of picodroplets per group increases linearly with *t*_open_ (*R*^2^=0.960). We note that for low picodroplet numbers (~10^2^), our standard curve results in a slight overestimate. At such low valve opening times, we approach the practical lower limit of microvalve actuation, which is set by the time required for mechanical deformation of the PDMS membrane valve. Nevertheless, this standard curve serves as a practical guideline in tuning our device to a desired dynamic range. Importantly, as we change the valve opening duration and therefore the number of picodroplets generated in our platform, we notice a minimal change in the average droplet transit time, which indicates high-volume monodispersity among our picodroplets (Fig. 5c). Likewise, the normalized average picodroplet fluorescence shows a minimal change among the different groups, indicating consistent concentration of fluorescent DNA within picodroplets (Fig. 5d). Notably, we observe negligible diffusion of fluorescently labeled DNA from droplets into the oil phase, as we have measured nearly identical average background fluorescence signals from the oil preceding each picodroplet group and the oil between picodroplets within a single picodroplet group(0.66 and 0.64 average photon counts, respectively). Importantly, we observe low variations in picodroplet number, volume, and fluorescence across multiple groups of picodroplets in multiple devices (*n* = 3), demonstrating that our platform has high reproducibility in generating picodroplets with monodisperse volume and uniform content at a preprogrammed dynamic range.

### Programmable contents in picodroplet groups

**Fig. 6:**
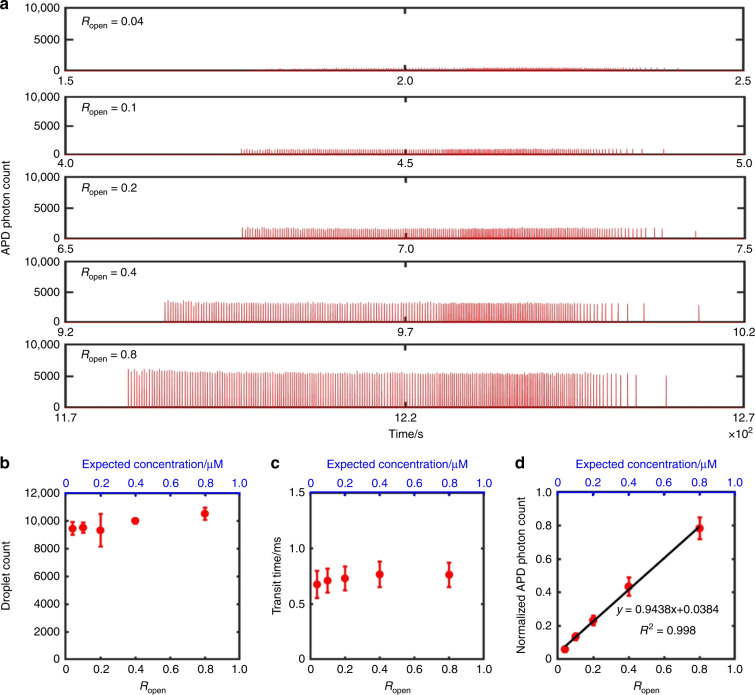
Customizable droplet contents in iPPA. **a** The fluorescence time trace of the five picodroplet groups with increasing *R*_open_ shows increasing fluorescence intensities. **b** The number of droplets generated remains approximately the same with increasing *R*_open_. **c** Droplet volume, characterized by the transit time of droplets, also remains approximately equal with increasing *R*_open_. **d** The normalized fluorescence intensities of picodroplet groups increase linearly with *R*_open_ (*R*^2^ = 0.998) with low experimental variability, demonstrating the reliability of iPPA in customizing droplet contents. All results were measured from three different experiments. The error bars in **b**–**d** depict ± 1 standard deviation

The ability to assemble customized combinations of nanoplugs in situ allows us to precisely program the contents of each picodroplet group in our iPPA platform. As a demonstration, we assembled five nanoplugs of equal volume, but increasing fluorescent DNA concentrations (0.04, 0.1, 0.2, 0.4, and 0.8 µM) by using increasing *R*_open_ (0.04, 0.1, 0.2, 0.4, and 0.8). These nanoplugs were then discretized into ~10000 picodroplets per group and finally measured at the detection channel. The time trace of droplet fluorescence clearly shows five well-separated picodroplet groups with distinct, increasing fluorescence intensities (Fig. 6a). As with Fig. 5a, for ease of viewing, we plot 1 in every 50 droplets from each droplet group in Fig. 6a. Importantly, only a very small fraction of the picodroplets in each group has noticeably higher or lower fluorescence intensities (Fig. S5). We note that while we generate only five distinct combinations of reagents and subsequent droplet groups to showcase the utility of our iPPA platform, our device has the capability of producing far more unique combinations by simply tuning the variables *R*_open_ and *t*_open_ or introducing additional reagents from additional inlets.

Our iPPA device allows us to precisely program droplet content while maintaining uniform picodroplet number and volume. From triplicate experiments, we plotted the number of picodroplets generated from each group against their respective *R*_open_. As expected, the numbers of droplets from each group remain approximately equal, since the *t*_open_ is kept constant across all groups (Fig. 6b). Likewise, picodroplet transit times remain approximately equal and vary minimally across different picodroplet groups, implying reproducible droplet volume and high monodispersity (Fig. 6c). Finally, the normalized fluorescence intensity of each picodroplet group increases linearly with *R*_open_ from triplicate experiments (Fig. 6d). The strong linearity (*R*^2^ = 0.998) and the small error bars (*n* = 3) clearly demonstrate the programmable customizability and reproducibility of our iPPA platform. These characteristics make our platform well suited for practical high-sensitivity and high-throughput analysis, especially for applications where analysis of samples against a panel of reagents/concentrations is required^35,36^.

## Discussion

In this work, we introduce iPPA, a fully integrated platform capable of programmable generation of picodroplets with tunable dynamic ranges and customized contents. Our iPPA device leverages the on-demand programmability of PDMS microvalves to assemble nanoplugs in situ with precise control of their sizes and contents. These nanoplugs are then discretized via flow focusing into highly monodisperse picodroplets. While each picodroplet group begins flowing through the on-chip incubation region, subsequent picodroplet groups are simultaneously assembled and then discretized. Full integration of nanoplug assembly, picodroplet generation, incubation, and detection in iPPA facilitates convenient, hands-free operation. We have herein demonstrated the ability of our platform in generating multiple picodroplet groups with a tunable dynamic range and customized composition with high reproducibility.

The iPPA platform offers practical advantages over traditional droplet platforms in picodroplet content customizability. Most traditional droplet devices lack the capability to precisely control the content within picodroplets for combinatorial analysis and therefore generate either homogeneous or random content in picodroplets. Multiple devices are usually required to produce picodroplets with different contents, and fluorometric coding or other complex indexing methods are necessary to demultiplex picodroplet groups that are mixed or pooled together for processing. iPPA addresses these challenges by allowing multiple customized reaction conditions to be programmatically assembled, discretized, and analyzed within a single device, without loss in performance across multiple groups. By employing a spatial (and hence, temporal) indexing scheme, we can easily distinguish signals from each picodroplet group without the need for complex optical indexing schemes. In this work, we demonstrate our platform’s capability by generating five distinct combinations of reagents using two inlets, but we note that our scalable device architecture has been previously used to assemble as many as 650 unique nanoplug combinations by taking advantage of 12 inlets within a single device^30,37–39^.

Current droplet platforms are limited to processing a finite and fixed number of droplets for a single reaction, often dictated by the particular application for which they were developed. iPPA provides the user with the flexibility to work with fewer or more droplets, depending on the desired dynamic range for the testing condition. For example, for applications where there exists an abundance of a target, in order to minimize redundant replicates and the associated reagent wastage, iPPA allows the user to work with a smaller number of droplets. On the other hand, for applications like single-bacteria studies where the target may be rare and where reaction results may vary due to cellular heterogeneity, more droplets can be generated and analyzed for more robust analysis of the target population.

Moreover, as a fully integrated system, iPPA automates every step from reaction assembly to discretization to incubation to detection. This obviates labor-intensive manual preparation of reaction mixtures and time-consuming user intervention between steps. These advantages help iPPA overcome crucial limitations of traditional droplet platforms and ultimately can help open the door for a vast array of combinatorial analysis applications.

We foresee several technical improvements that can help iPPA further push the limits of throughput. By minimizing the spacing between adjacent picodroplet groups, we can maximize the number of distinct picodroplet groups processed per unit time. This may be accomplished by employing an immiscible separation plug^26^ or by reducing Taylor dispersion of droplets within the incubation channel^40^. Furthermore, we may reduce the space between subsequent nanoplugs by shortening the mixing channel via on-chip chaotic mixing structures^41^. With potential optimizations in place, our iPPA platform can become a useful tool for practical combinatorial analysis applications in clinical laboratories as well as industry.

## Materials and methods

### Device design

Our iPPA device is composed of two distinct layers—a fluidic layer where nanoplugs and picodroplets are generated, incubated, and detected, and a valve layer that controls the creation and movement of these nanoplugs and picodroplets. The fluidic layer consists of 11 distinct inlet and outlet ports, including two sample inlet ports, four reagent inlet ports, two oil inlet ports (for nanoplug assembly and picodroplet generation, respectively), two pressure-release outlets, and one final device outlet port. All sample and reagent inlets feed into a common central channel, where nanoplugs are assembled. The central channel extends into a serpentine mixing channel that is 200-µm wide and 14-cm long. The mixing channel narrows into a 10-µm-wide constriction that intersects a pair of orthogonal channels that carry picodroplet generation oil. This intersection forms a flow-focusing junction for discretizing nanoplugs into picodroplets. Immediately downstream of the junction, the channel gradually widens and feeds into a serpentine incubation channel that is 500-μm wide and 75-cm long. At the end, the incubation channel narrows into a 10-µm-wide detection constriction before finally widening again and leading into the device outlet port.

The device’s valve control layer consists of 10 microvalves for precise control of nanoplug assembly and picodroplet generation. Of the 10 microvalves, one microvalve controls the nanoplug assembly oil inlet, two microvalves control the sample inlet, four microvalves control the four reagent inlets, two microvalves control the pressure-release outlets, and one isolation valve regulates nanoplug entry into the flow-focusing junction. All microvalves are 600-µm long and 200-µm wide, and they fully span the full width of the channels they control.

### Master mold fabrication

Masks for the fluidic and valve control layers were designed in L-Edit v16.0 (Tanner EDA, Monrovia, CA, USA). Mask patterns were printed onto high-quality transparencies at 20,000 dpi by CAD/Art Services, Inc. (Bandon, OR, USA). Master molds for both fluidic and valve control layers of the device were microfabricated via standard photolithography on 4-inch silicon wafers (Polishing Corporation of America, Santa Clara, CA, USA). To construct the mold for the fluidic channel, SPR-220-7 (positive photoresist; Microchem Corp., Newton, MA, USA) was initially spin-coated to a height of ~20 µm. This positive photoresist was used to fabricate segments of the fluidic channels that intersect the PDMS microvalves. The rounded cross-section of SPR-220-7 photoresist allows the microfluidic channels to be effectively closed off by the PDMS membrane upon microvalve actuation. For fabricating the remaining fluidic channels that do not intersect PDMS microvalves (e.g., mixing and incubation channels), SU8-3050 (Microchem Corp., Newton, MA, USA) negative photoresist was spin-coated to a height of ~60 µm and patterned via photolithography. The SU-8 and SPR-220-7 channels were aligned together using common alignment marks on the wafer. For the valve control mold, a single layer of SU8-3025 (Microchem Corp., Newton, MA, USA) negative photoresist was spun to a height of ~30 µm and patterned via photolithography.

### Microfluidic device fabrication

iPPA devices were fabricated with polydimethylsiloxane (PDMS) via multilayer soft lithography^30,31,37,39^. Our devices employ a “push-up” architecture, wherein the valve control layer is sandwiched between the fluidic layer and a glass slide. This architecture enables channels to be completely surrounded by hydrophobic PDMS, thereby preventing fluids from coming into contact with and sticking to the hydrophilic glass surface.

Device fabrication was initiated by silanizing all molds with chlorotrimethylsilane (Sigma-Aldrich, St. Louis, MO, USA) for at least 15 min to minimize adhesion between PDMS and the photoresist structures. For each device, a thick fluidic layer (~5 mm) was fabricated by pouring 49.5 g of 10:1 (base to curing agent ratio) PDMS (SYLGARD 184 Silicone Elastomer Kit, Dow Corning, Midland, MI, USA) onto the fluidic mold and curing for 30 min at 80 °C. Cured PDMS was then peeled from the mold and holes were punched through the inlet and outlet ports with a sharpened needle (20 gauge; McMaster-Carr, Elmhurst, IL, USA). Next, a thin valve control layer (~50 μm) was fabricated by spin-coating 15:1 PDMS onto the valve control mold at 1000 rpm and curing for 12 min at 80 °C. In order to effectively peel the thin PDMS valve control layer from its mold, a sacrificial sheet of PDMS was used as a structural support. The sacrificial PDMS sheet was formed by spin-coating 6:1 PDMS on a blank wafer at 100 rpm and curing at 80 °C for 7 min. This sheet was then peeled and reversibly bonded to the top of the valve control layer by heating at 80 °C for 5 min. The valve-control-sacrificial-sheet compound layer was peeled from the valve control mold, holes were punched through valve inlet ports, and the compound layer was permanently bonded to a cover glass slide (75 mm in length, 50 mm in width, and 130 μm in thickness, Ted Pella, Redding, CA) using oxygen plasma treatment (42 W, 500 mTorr, 45 s). Next, the sacrificial PDMS sheet was peeled and released from the valve control layer. Finally, the thick fluidic layer was permanently bonded to the valve control layer via oxygen plasma treatment (42 W, 500 mTorr, 45 s) after manual alignment using a stereoscope. The completed device was baked for at least 24 h at 80 °C. In order to ensure optimal hydrophobicity, all channels of the chip were coated with a commercial hydrophobic coating agent (Rain-X^®^ Original, ITW Global Brands, Houston, TX, USA)^41^ followed by baking at 80 °C for 1 h to minimize liquid residues from sticking to the sample inlet channels.

### Device operation

Nanoplugs in our device were assembled by programmatic actuation of on-chip microvalves. A set of solenoid valves (Pneumadyne, Plymouth, Minnesota, USA) was used to open and close individual microvalves in the microfluidic device. Connections between solenoid valves and microvalves were established using water-filled Tygon microbore tubing (0.02-inch ID and 0.06-inch OD; Cole-Parmer, Vernon Hills, IL, USA) connected to 23-gauge blunt needles (McMaster-Carr) inserted at designated valve inlet ports. A pressure of 30 psi was applied to facilitate valve closure. Unique sequences of valve open/close timings were programmed and controlled by custom software written in MATLAB (MathWorks, Natick, MA, USA)^30^. Nanoplugs of unique volume and content were assembled by controlling the opening times of the microvalves regulating flow from the sample and reagent inlets.

The continuous phase oil used to assemble nanoplugs (i.e., nanoplug assembly oil) consisted of a fluorinated oil FC-40 (3M, Two Harbors, MN, USA) and a nonionic fluorous-soluble surfactant 1H, 1H, 2H, 2H-Perfluoro-1-octanol (PFO; Sigma-Aldrich) (4:1 v/v). The oil used to generate picodroplets (i.e., picodroplet generation oil) was purchased from BioRad (QX200™ Droplet Generation Oil for EvaGreen, Hercules, CA, USA). Both oils were initially loaded into Tygon microbore tubing and connected to the iPPA device via their designated inlet. We used a 1 μM solution of Alexa Fluor 546-labeled single-stranded DNA (Integrated DNA Technologies Inc., Coralville, Iowa, USA) and phosphate-buffered saline (PBS) (Quality Biological, Gaithersburg, MD, USA) as surrogates for samples and reagents. To initiate loading, 5 µL or more of the sample and reagent solutions were individually drawn into PTFE tubing (30 AWG; Cole-Parmer) and directly connected to our device’s sample and reagent inlets, respectively. Before assembling any nanoplugs, the nanoplug assembly oil was injected to fill and prime the central channel of the device, while the picodroplet generation oil filled the incubation and detection regions of the device. During device operation, all the inlets of the device were kept under constant pressure, with distinct optimized input pressures for the nanoplug assembly oil (5.2 psi), picodroplet generation oil (3.8 psi), samples (1.7 psi), and reagents (1.7 psi). By pneumatically pressurizing the samples/reagents into our device, there is negligible samples loss and dead volume in our device.

Discretization of nanoplugs into picodroplets was achieved by pushing nanoplugs past the mixing channel into the flow-focusing junction. The isolation valve was opened during picodroplet generation, and the pressurized picodroplet generation oil could passively discretize the incoming nanoplug into hundreds to tens of thousands of picodroplets with a generation frequency of ~8000 droplets per minute. Following this step, the droplets continued to flow into the incubation, detection, and outlet channels, propelled by the pressurized picodroplet generation oil.

### Data acquisition and analysis

Nanoplug assembly, mixing, and picodroplet generation were observed with an inverted microscope (IX71; Olympus Corp., Tokyo, Japan) with either a ×1.25 magnification objective lens (Olympus PlanAPO N ×1.25/0.04 NA) or a ×4 magnification objective lens (Olympus UPlanFl ×4/0.13 NA). A digital single-lens reflex (DSLR) camera (EOS 60D; Canon, Inc., Tokyo, Japan) that was mounted on our microscope and interfaced via EOS Utility software (Canon U.S.A., Inc., Melville, NY 11747) was used to take images and videos of device operation. Individual screenshots of relevant regions were taken from videos via the multimedia player software iMovie (Apple Inc., Cupertino, CA). Image J was used for analysis and processing of all bright-field images^42^. The diameter of picodroplets was calculated by measuring their width in pixels and comparing with the fixed width of the incubation channel (500 μm).

Fluorescence in nanoplugs and picodroplets was measured by a custom-built laser-induced fluorescence (LIF) system. This system included a 552-nm laser excitation source (OBIS, Coherent, Inc.) and a silicon avalanche photodiode detector (APD) (SPCM-AQRH13, ThorLabs). The laser was operated at 3-mW power and was focused into the detection constriction of the device using a ×40 objective (Thorlabs RMS40X-PF, NA 0.75, focal depth ~0.6 μm). As nanoplugs and picodroplets flowed through the detection constriction, fluorescence emitted by nanoplugs and picodroplets was continuously acquired using the APD with 0.1-ms sampling time and recorded by a custom LabVIEW program.

## Supplementary information

Supplementary Information_Revised

Customizing Droplet Contents and Dynamic Ranges via Integrated Programmable Picodroplet Assembler

## References

[1] Guo MT, Rotem A, Heyman JA, Weitz DA (2012). Droplet microfluidics for high-throughput biological assays. Lab Chip.

[2] Sesen M, Alan T, Neild A (2017). Droplet control technologies for microfluidic high throughput screening (μHTS). Lab Chip.

[3] Kaushik Aniruddha M., Hsieh Kuangwen, Wang Tza-Huei (2018). Droplet microfluidics for high-sensitivity and high-throughput detection and screening of disease biomarkers. Wiley Interdisciplinary Reviews: Nanomedicine and Nanobiotechnology.

[4] Simmons A, Schultz DM (2012). Analog-to-digital Primed for resistance. Nature.

[5] Miller OJ (2012). High-resolution dose – response screening using droplet-based microfluidics. Proc. Natl Acad. Sci. USA.

[6] Agresti JJ (2010). Ultrahigh-throughput screening in drop-based microfluidics for directed evolution. Proc. Natl Acad. Sci. USA.

[7] Mazutis L (2013). Single-cell analysis and sorting using droplet-based microfluidics. Nat. Protoc..

[8] Li L (2006). Nanoliter microfluidic hybrid method for simultaneous screening and optimization validated with crystallization of membrane proteins. Proc. Natl Acad. Sc. USA.

[9] Pekin D (2011). Quantitative and sensitive detection of rare mutations using droplet-based microfluidics. Lab Chip.

[10] Zec H, Shin DJ, Wang TH (2014). Novel droplet platforms for the detection of disease biomarkers. Expert Rev. Mol. Diagn..

[11] Derzsi L, Kaminski TS, Garstecki P (2016). Antibiograms in five pipetting steps: precise dilution assays in sub-microliter volumes with a conventional pipette. Lab Chip.

[12] Kaushik AM (2017). Accelerating bacterial growth detection and antimicrobial susceptibility assessment in integrated picoliter droplet platform. Biosens. Bioelectron..

[13] Zhang Y, Shin DJ, Wang TH (2013). Serial dilution via surface energy trap-assisted magnetic droplet manipulation. Lab Chip.

[14] Rane TD, Zec HC, Puleo C, Lee AP, Wang TH (2012). Droplet microfluidics for amplification-free genetic detection of single cells. Lab Chip.

[15] Taly V, Pekin D, Abed AEl, Laurent-Puig P (2012). Detecting biomarkers with microdroplet technology. Trends Mol. Med..

[16] Teh SY, Lin R, Hung LH, Lee AP (2008). Droplet microfluidics. Lab Chip.

[17] Whale AS, Huggett JF, Tzonev S (2016). Fundamentals of multiplexing with digital PCR. Biomol. Detect. Quantif..

[18] Hindson BJ (2011). High-throughput droplet digital PCR system for absolute quantitation of DNA copy number. Anal. Chem..

[19] Zhong Q (2011). Multiplex digital PCR: Breaking the one target per color barrier of quantitative PCR. Lab Chip.

[20] Taly V (2013). Multiplex picodroplet digital PCR to detect KRAS mutations in circulating DNA from the plasma of colorectal cancer patients. Clin. Chem..

[21] Abate AR, Hung T, Mary P, Agresti JJ, Weitz DA (2010). High-throughput injection with microfluidics using picoinjectors. Proc. Natl. Acad. Sci..

[22] O’Donovan B, Eastburn DJ, Abate AR (2012). Electrode-free picoinjection of microfluidic drops. Lab Chip.

[23] Chen CH (2013). Multiplexed protease activity assay for low-volume clinical samples using droplet-based microfluidics and its application to endometriosis. J. Am. Chem. Soc..

[24] Mazutis L, Baret JC, Griffiths AD (2009). A fast and efficient microfluidic system for highly selective one-to-one droplet fusion. Lab Chip.

[25] Mazutis L, Griffiths AD (2012). Selective droplet coalescence using microfluidic systems. Lab Chip.

[26] Kaminski TS, Jakiela S, Czekalska MA, Postek W, Garstecki P (2012). Automated generation of libraries of nL droplets. Lab Chip.

[27] Chang JC, Swank Z, Keiser O, Maerkl SJ, Amstad E (2018). Microfluidic device for real-time formulation of reagents and their subsequent encapsulation into double emulsions. Sci. Rep.

[28] Guo F (2010). Valve-based microfluidic device for droplet on-demand operation and static assay.. Appl. Phys. Lett..

[29] Zeng S, Li B, Su X, Qin J, Lin B (2009). Microvalve-actuated precise control of individual droplets in microfluidic devices. Lab Chip.

[30] Zec H, Rane TD, Wang TH (2012). Microfluidic platform for on-demand generation of spatially indexed combinatorial droplets. Lab Chip.

[31] Unger MA, Chou HP, Thorsen T, Scherer A, Quake SR (2000). Monolithic microfabricated valves and pumps by multilayer soft lithography. Science.

[32] Xia Y, Whitesides GM (1998). Soft lithography. Angew. Chem.-Int. Ed..

[33] Song H, Bringer MR, Tice JD, Gerdts CJ, Ismagilov RF (2003). Experimental test of scaling of mixing by chaotic advection in droplets moving through microfluidic channels. Appl. Phys. Lett..

[34] Frenz L, Blank K, Brouzes E, Griffiths AD (2009). Reliable microfluidic on-chip incubation of droplets in delay-lines. Lab Chip.

[35] Shen F (2011). Multiplexed quantification of nucleic acids with large dynamic range using multivolume digital RT-PCR on a rotational SlipChip tested with HIV and hepatitis C viral load. J. Am. Chem. Soc..

[36] Kreutz JE (2011). Theoretical design and analysis of multivolume digital assays with wide dynamic range validated experimentally with microfluidic digital PCR. Anal. Chem..

[37] Rane TD, Zec HC, Wang TH (2015). A barcode-free combinatorial screening platform for matrix metalloproteinase screening. Anal. Chem..

[38] Hsieh K, Zec HC, Ma PC, Rane TD, Wang TH (2015). Enhancing throughput of combinatorial droplet devices via droplet bifurcation, parallelized droplet fusion, and parallelized detection. Micromachines.

[39] Zec HC (2018). Programmable microfluidic genotyping of plant DNA samples for marker-assisted selection. Microsyst. Nanoeng..

[40] Stroock AD (2002). Chaotic mixer for microchannels. Sci..

[41] Axt, B., Hsieh, Y. F., Nalayanda, D. & Wang, T. H. Impedance feedback control of microfluidic valves for reliable post processing combinatorial droplet injection. *Biomed. Microdevices* **19**, 61 (2017). 10.1007/s10544-017-0203-2 28681238

[42] Schneider CA, Rasband WS, Eliceiri KW (2012). NIH Image to ImageJ: 25 years of image analysis. Nat. Methods.

