# Supplementary material for: Customizing droplet contents and dynamic ranges via integrated programmable picodroplet assembler
Source: Microsyst Nanoeng. 2019 Jul 1;5:22. doi: 10.1038/s41378-019-0062-5 (PMC6799804; doi:10.1038/s41378-019-0062-5)
Supplement: Supplementary file 1 — Supplementary Information_Revised [file 41378_2019_62_MOESM1_ESM.docx]

Supporting Information

Customizing Droplet Contents and Dynamic Ranges via Integrated Programmable Picodroplet Assembler

Pengfei Zhang, Aniruddha Kaushik, Kuangwen Hsieh, and Tza-Huei “Jeff” Wang*


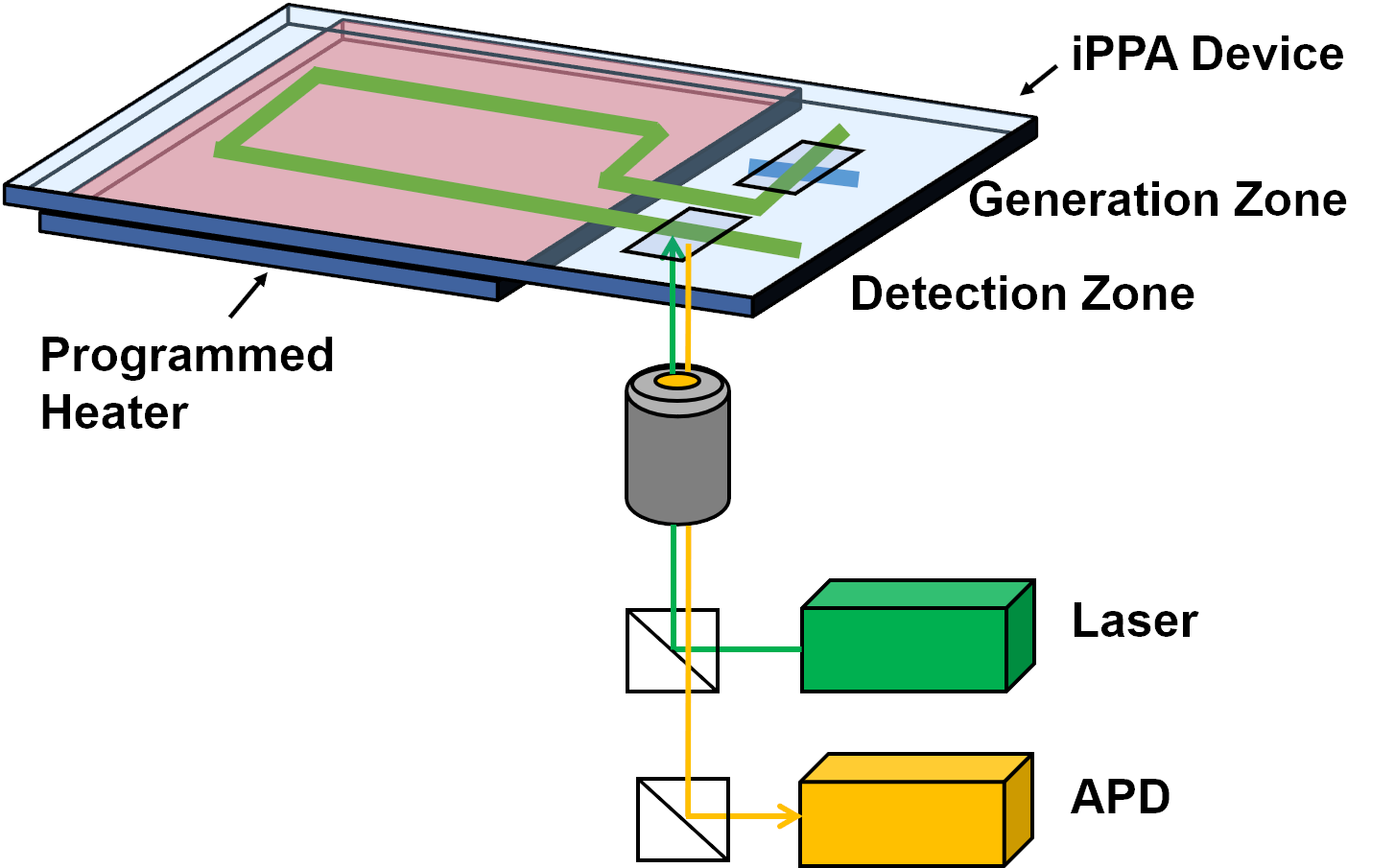


Figure S1. Schematic of the incubation and detection system for the integrated programmable picodroplet assembler (iPPA). For incubation, a programmed heater is used to heat the iPPA’s incubation channel with controlled temperature on demand. Following droplet incubation, picodroplets were detected using the laser-induced-fluorescence microscope. The fluorescence intensity of droplets was measured using a silicon avalanche photodiodes detector (APD).


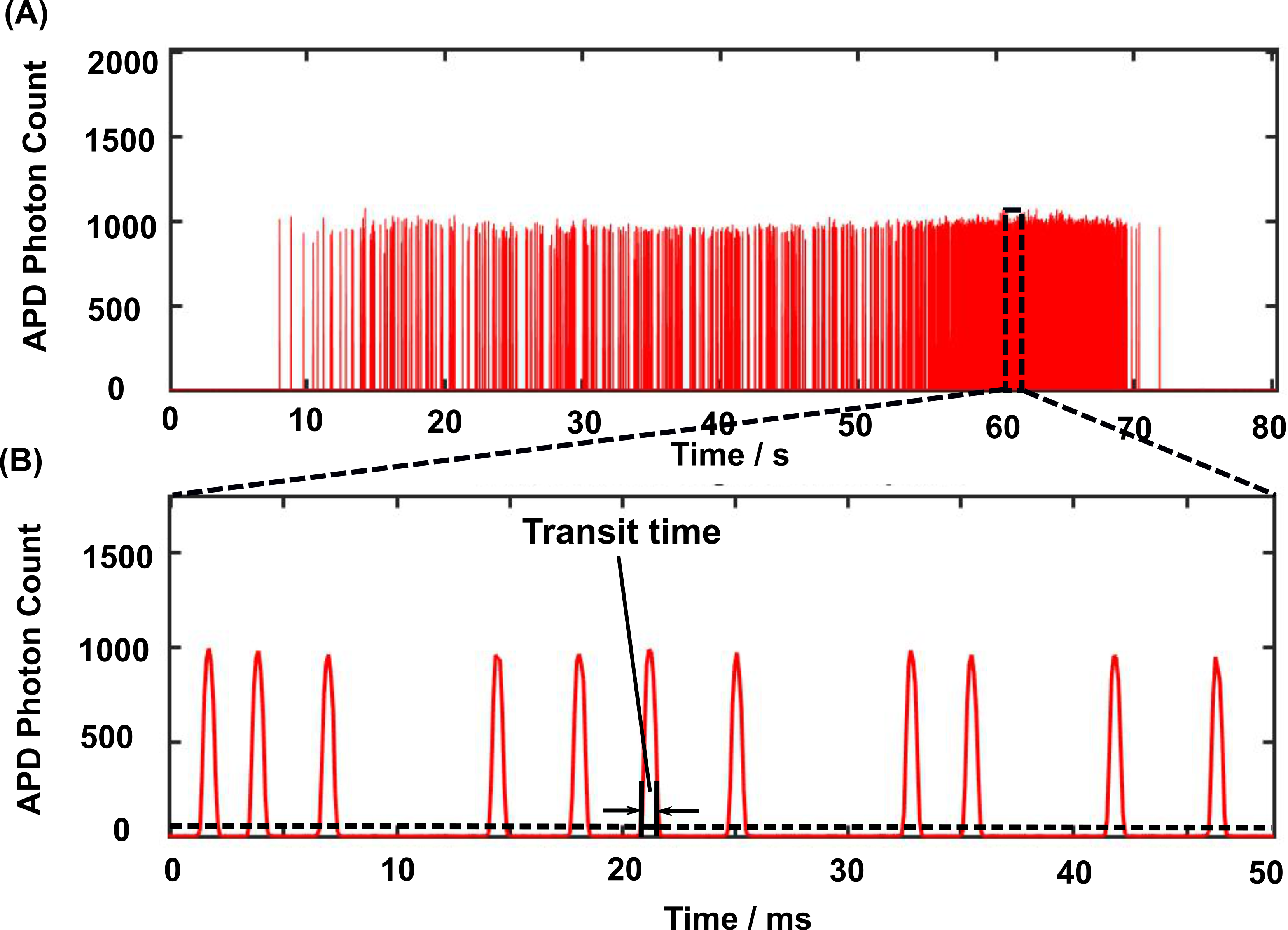


Figure S2. Time trace of picodroplet fluorescence. (A) Fluorescence from picodroplets traversing the detection region of our device is measured by our custom LIF detector and plotted against time. (B) The width of picodroplet peaks, or the transit time of picodroplets flowing through detection point, is used to characterize droplet volume. APD photon count indicates the number of incident photons detected per 0.1 ms by a silicon avalanche photodiode detector.


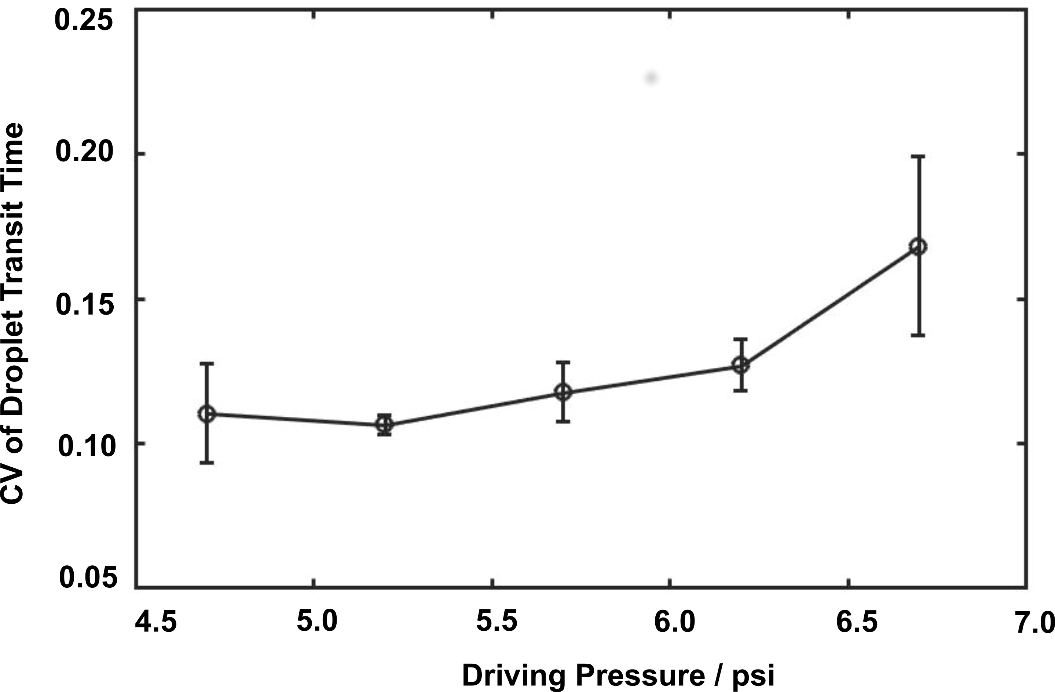


Figure S3. Optimizing driving pressure of nanoplugs. As a measure of monodispersity, CV (standard deviation/mean) of droplet transit time is plotted for 5 distinct picodroplet groups generated at 5 different driving pressures. At 5.2 psi, we generate the most monodisperse picodroplet group with the least chip-to-chip variation (n=3).


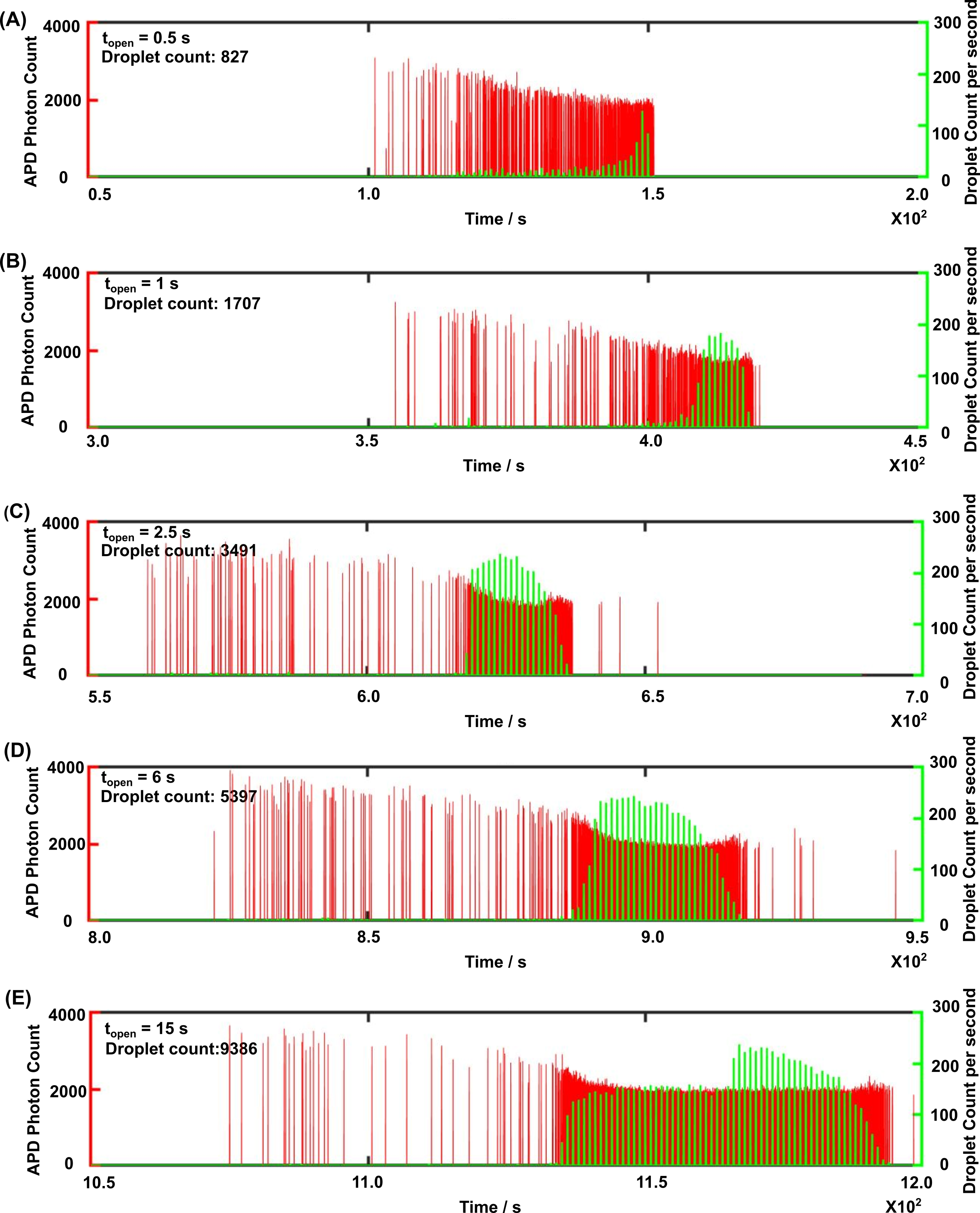


Figure S4. Raw time trace of droplet fluorescence represents mostly uniform fluorescence intensity in each group. (A) - (E) Left: The droplet fluorescence for each group is measured by our custom LIF detector and plotted against time individually. (A) – (E) Right: droplet number detected in each second was plotted to show that only small fraction of picodroplets in each group shows noticeably higher fluorescence. The droplet number in each group increases because of increased t_open_. APD photon count indicates the number of incident photons detected per 0.1 ms by a silicon avalanche photodiode detector.


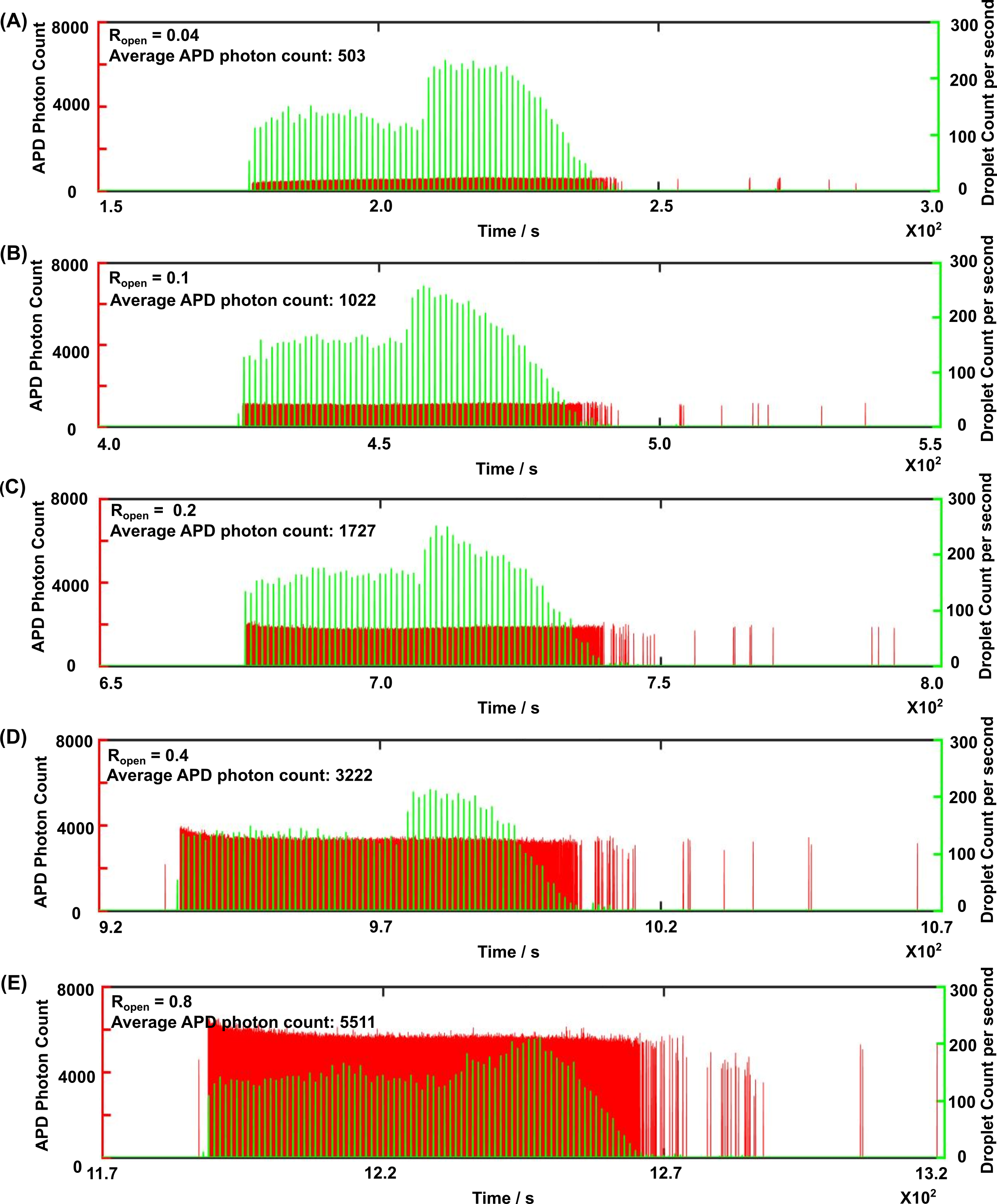


Figure S5. Extended time trace of droplet fluorescence and uniform signal intensity in picodroplet groups with different concentrations. (A) - (E) Left: The droplet fluorescence in each group is measured by our custom LIF detector and plotted against time individually. (A) – (E) Right: droplet counts detected in each signal were plotted to show that only small fraction of droplets in each group shows noticeable higher or lower fluorescence. The droplet fluorescence intensity increases gradually due to higher R_open_ value. APD photon count indicates the number of incident photons detected per 0.1 ms by a silicon avalanche photodiode detector.
