# Supplementary material for: Customizing droplet contents and dynamic ranges via integrated programmable picodroplet assembler
Source: Microsyst Nanoeng. 2019 Jul 1;5:22. doi: 10.1038/s41378-019-0062-5 (PMC6799804; doi:10.1038/s41378-019-0062-5)
Supplement: Supplementary file 2 — Customizing Droplet Contents and Dynamic Ranges via Integrated Programmable Picodroplet Assembler [file 41378_2019_62_MOESM2_ESM.pdf]

# Supporting Information

## Customizing Droplet Contents and Dynamic Ranges via Integrated Programmable Picodroplet Assembler

*Pengfei Zhang, Aniruddha Kaushik, Kuangwen Hsieh, and Tza-Huei "Jeff" Wang**

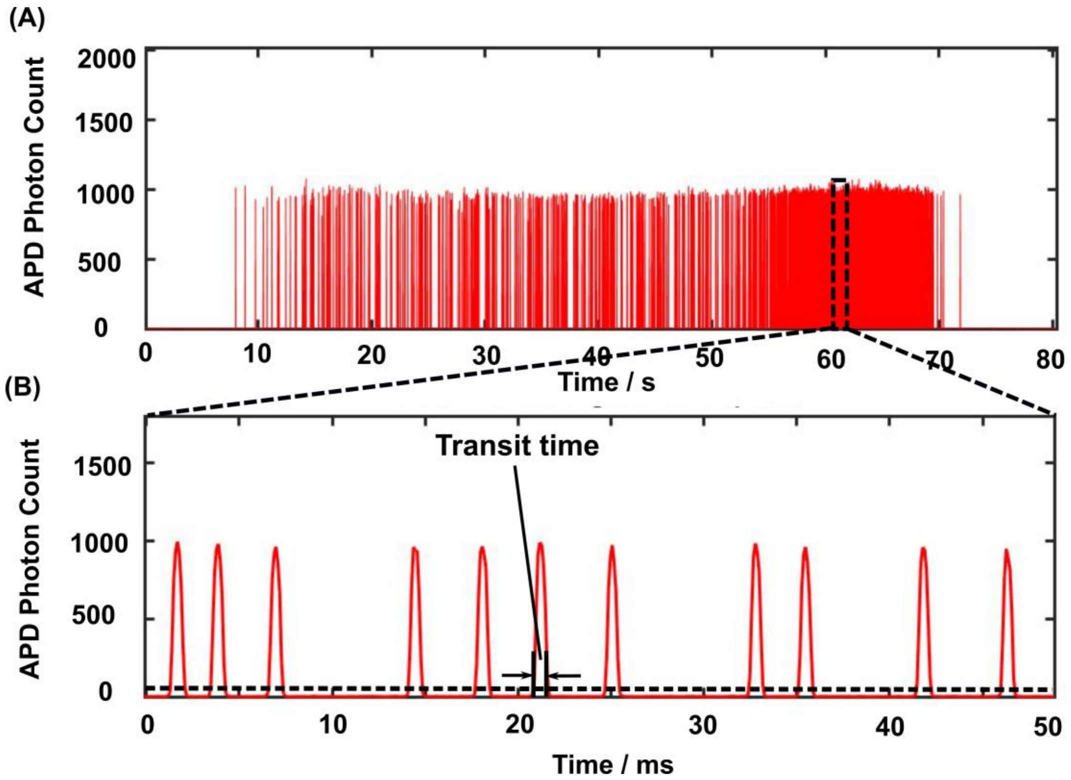


Figure S1. Time trace of picodroplet fluorescence. (A) Fluorescence from picodroplets traversing the detection region of our device is measured by our custom LIF detector and plotted against time. (B) The width of picodroplet peaks, or the transit time of picodroplets flowing through detection point, is used to characterize droplet volume

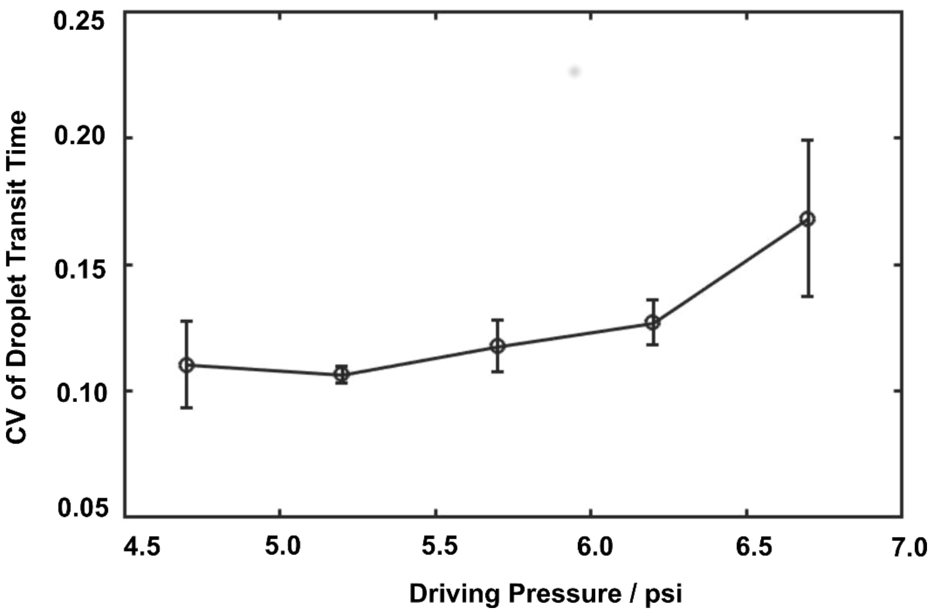


Figure S2. Optimizing driving pressure of nanoplugs. As a measure of monodispersity, CV (standard deviation/mean) of droplet transit time is plotted for 5 distinct picodroplet groups generated at 5 different driving pressures. At 5.2 psi, we generate the most monodisperse picodroplet group with the least chip-to-chip variation (n=3).

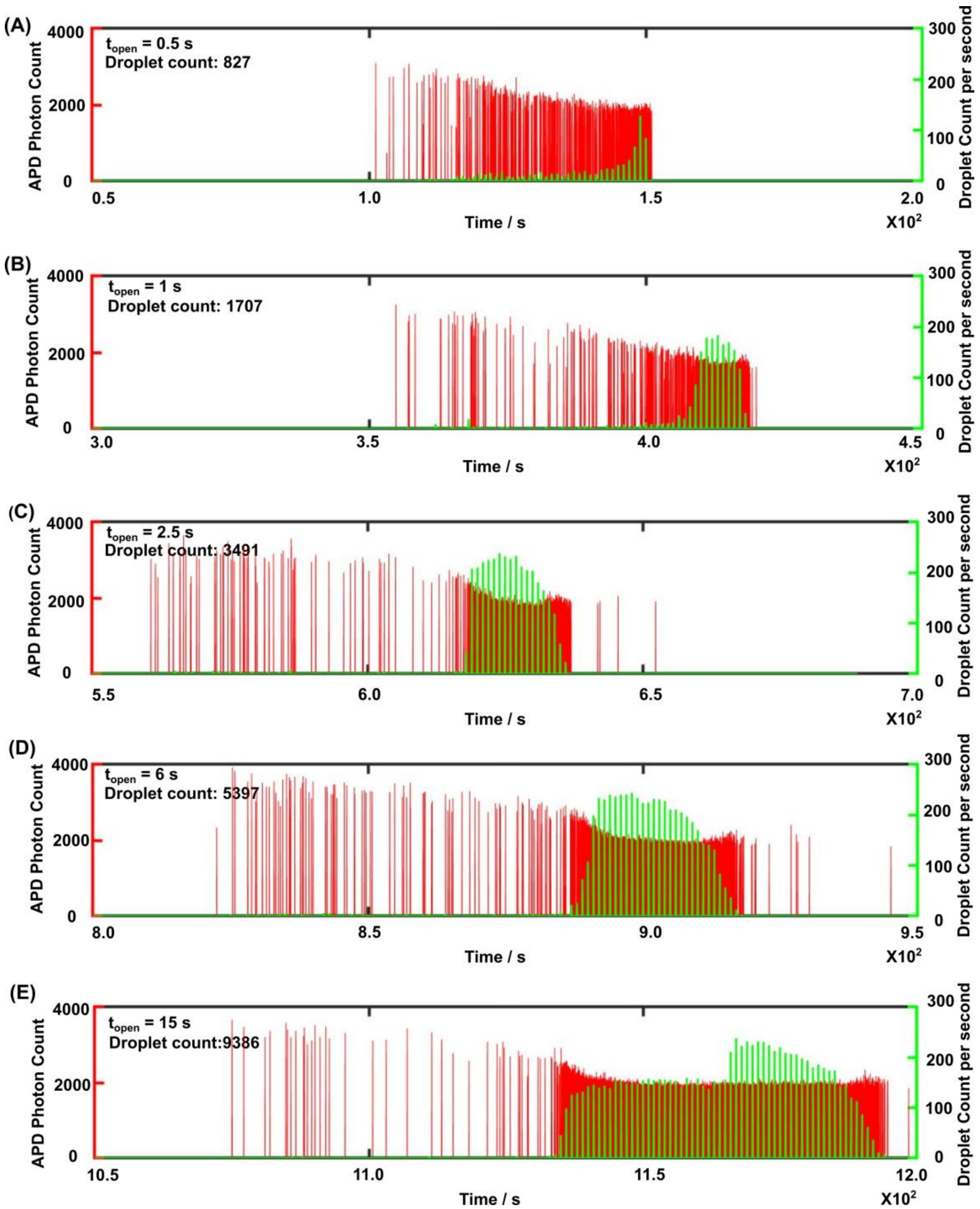


Figure S3. Raw time trace of droplet fluorescence represents mostly uniform fluorescence intensity in each group. (A) - (E) Left: The droplet fluorescence for each group is measured by our custom LIF detector and plotted against time individually. (A) – (E) Right: droplet number detected in each second was plotted to show that only small fraction of picodroplets in each group shows noticeably higher fluorescence. The droplet number in each group increases because of increased $t_{open}$.

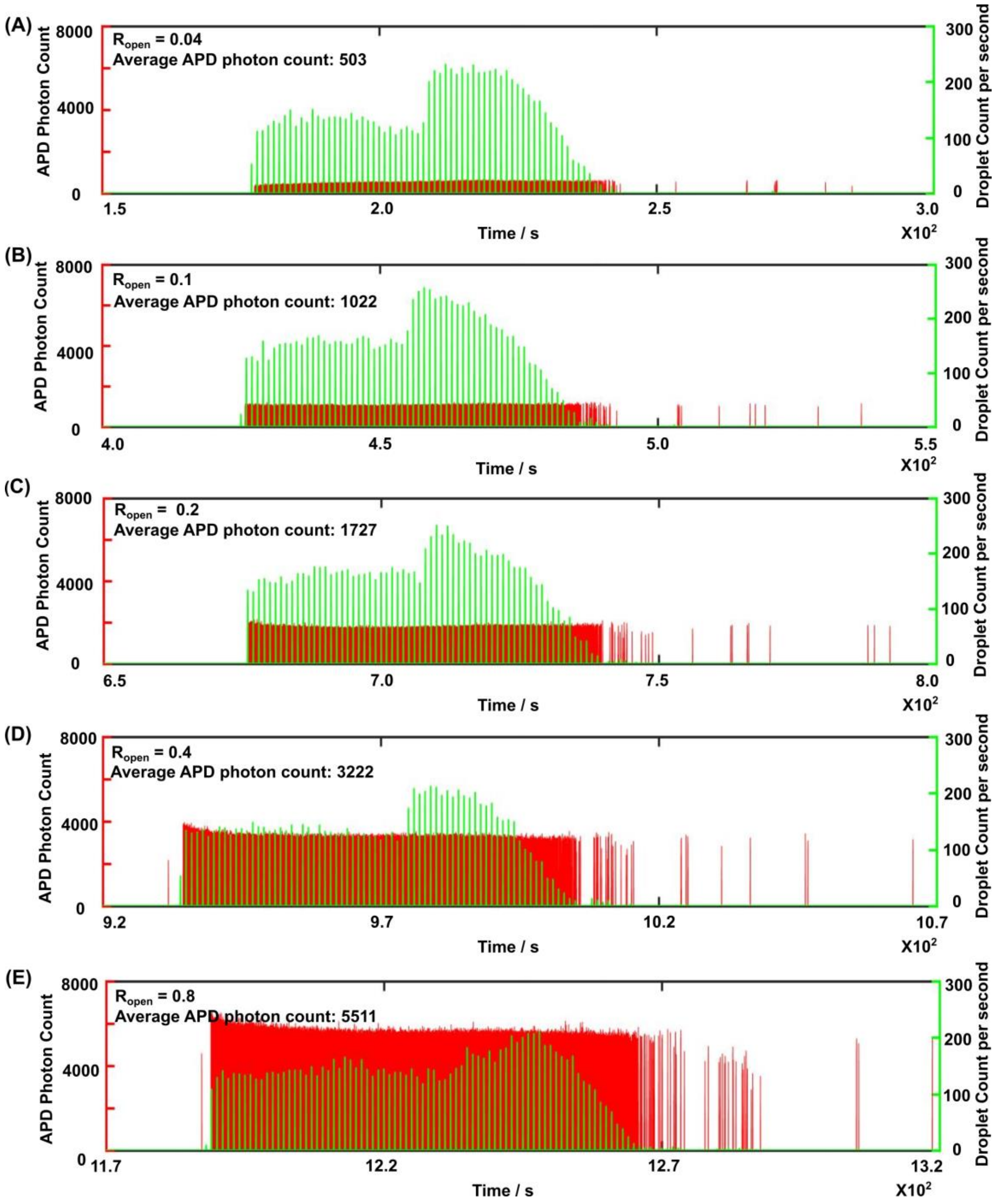


Figure S4. Extended time trace of droplet fluorescence and uniform signal intensity in picodroplet groups with different concentrations. (A) - (E) Left: The droplet fluorescence in each group is measured by our custom LIF detector and plotted against time individually. (A) – (E) Right: droplet counts detected in each signal were plotted to show that only small fraction of droplets in each group shows noticeable higher or lower fluorescence. The droplet fluorescence intensity increases gradually due to higher $R_{open}$ value
